# Pharmacogenomics and risk stratification in cardiovascular care: Insights from randomized controlled trials

**DOI:** 10.1097/MD.0000000000042868

**Published:** 2025-06-13

**Authors:** Sajjad Ahmed Khan, Sadab Khan, Huma Kausar, Prashim Sharma, Alisha Adhikari, Surya Bahadur Parajuli, Madhab Bista

**Affiliations:** a Birat Medical College Teaching Hospital, Morang, Nepal; b Karnali Academy of Health Sciences, Nepal; c Department of Community Medicine, Birat Medical College Teaching Hospital, Nepal; d Department of Cardiovascular Medicine, Birat Medical College Teaching Hospital, Nepal.

**Keywords:** cardiology, genomics, personalized medicine

## Abstract

Genomics and personalized medicine are transforming cardiology by improving the assessment, diagnosis, and treatment of cardiovascular diseases (CVDs). By understanding the genetic factors underlying CVDs, personalized treatment strategies can be developed to enhance patient outcomes and reduce the burden of cardiovascular conditions. The integration of genomics into clinical practice enables more precise risk assessments, early detection, and targeted interventions. This narrative review examines randomized clinical trials (RCTs) on genotype-guided therapies, pharmacogenomics, and personalized medicine in cardiology. Studies were selected for their contributions to identifying genetic factors influencing CVD risk, evaluating pharmacogenomic data, and exploring personalized treatment approaches. Key outcomes analyzed include treatment efficacy, genetic associations, and clinical implications of genetic insights in cardiovascular care. The findings highlight the identification of genetic variants linked to increased CVD risk and the development of genomic risk scores for personalized risk prediction. Pharmacogenomic studies also revealed genetic determinants of drug response, particularly in cardiovascular treatments. Genetic testing has shown promise in guiding antiplatelet therapy selection, drug dosage optimization, and minimizing adverse effects, thus improving treatment efficacy. Despite the potential of genomics and personalized medicine, challenges such as clinical implementation, cost-effectiveness, and ethical concerns persist. To fully benefit from personalized cardiovascular care, future research should focus on validating genetic markers, refining risk prediction models, and overcoming barriers to widespread adoption. With continued development, these approaches could revolutionize cardiovascular care, providing more effective, individualized treatments and improving patient outcomes.

## 1. Introduction

Cardiovascular diseases (CVDs) pose a significant global health burden, with high morbidity and mortality rates.^[1]^ Despite advancements in treatment modalities and risk factor management, the complex interplay of genetic, environmental, and lifestyle factors contributes to the heterogeneous nature of CVDs, necessitating personalized approaches to prevention and treatment.^[2]^ In recent years, there has been a growing recognition of the potential of genomics and personalized medicine to revolutionize the field of cardiology by elucidating the genetic underpinnings of CVDs and tailoring interventions to individual patients.^[3]^

The advent of genome-wide association studies has enabled the identification of numerous genetic variants associated with CVD risk, providing insights into the underlying pathophysiology of conditions such as coronary artery disease (CAD), heart failure (HF), and arrhythmias.^[4,5]^ Furthermore, advances in next-generation sequencing technologies have facilitated the discovery of rare genetic mutations and copy number variations implicated in familial forms of CVDs.^[6]^ These genomic discoveries have not only enhanced our understanding of disease mechanisms but also hold promise for the development of novel diagnostic and therapeutic strategies.

Personalized medicine in cardiology encompasses a wide range of approaches aimed at tailoring medical care to individual patients based on their genetic makeup, phenotypic characteristics and environmental influences.^[7]^ Genomic risk scores, computed based on an individual’s genetic variants, have emerged as valuable tools for predicting an individual’s susceptibility to CVDs and stratifying patients according to their risk profile.^[8]^ Additionally, pharmacogenomic studies have shed light on the variability in drug response and adverse effects observed in cardiovascular pharmacotherapy, highlighting the importance of considering genetic factors in treatment selection and dosing.^[9]^ Despite the promise of genomics and personalized medicine in cardiology, several challenges remain, including the interpretation of genetic variants of uncertain significance, issues related to data privacy and security, and disparities in access to genetic testing and specialized care.^[10]^ Moreover, the clinical utility and cost-effectiveness of genomic testing in improving cardiovascular outcomes warrant further investigation.^[11]^

In this review, we aim to synthesize the current evidence on genomics and personalized medicine in cardiology, including the identification of genetic markers, development of genomic risk scores, pharmacogenomic insights, and clinical implications for personalized treatment strategies. By critically evaluating the existing literature, we seek to elucidate the potential impact of genomics and personalized medicine on reshaping the landscape of cardiovascular care and inform future research directions in the field.

## 2. Materials and methods

### 2.1. Search strategy

A comprehensive literature search was conducted to identify relevant studies focusing on genomics and personalized medicine in cardiology. The search strategy included a combination of keywords and Medical Subject Headings terms related to “genomics,” “personalized medicine,” and “cardiology,” with no language restrictions applied. Additionally, reference lists of included studies and relevant review articles were hand-searched to identify additional potentially eligible studies.

### 2.2. Study selection

Inclusion criteria: Randomized clinical trials (RCTs) published in peer-reviewed journals focused on genetic markers, genomic risk scores, pharmacogenomics, or personalized treatment strategies in CVDs, providing sufficient data for analysis.

Exclusion criteria: Studies were excluded if they were review articles, case reports, or conference abstracts.

### 2.3. Data extraction

Two independent reviewers screened the titles and abstracts of identified articles to assess their eligibility for inclusion. Full-text articles of potentially eligible studies were retrieved and assessed for final inclusion based on predefined criteria. Any discrepancies between reviewers were resolved through discussion and consensus. Data extraction was performed using a standardized data extraction form, including information on study characteristics (e.g., study design, sample size), participant demographics, genetic markers or interventions studied, outcomes measured, and key findings.

### 2.4. Quality assessment

The quality of included studies was evaluated using the Cochrane risk of bias tool. Quality assessment was performed independently by 2 reviewers, with any disagreements resolved through discussion and consultation with a 3rd reviewer if necessary.

### 2.5. Data synthesis

Data synthesis involved a narrative summary of study findings, organized according to the key themes identified in the included studies. Quantitative data, such as effect sizes and measures of variability, were extracted and synthesized where appropriate. Meta-analysis was considered if there were a sufficient number of comparable studies reporting similar outcomes. However, due to the anticipated heterogeneity of study designs and outcomes, a qualitative synthesis was deemed more appropriate for this review.

### 2.6. Ethical considerations

This review did not involve human subjects and utilized only publicly available data from previously published studies. Therefore, ethical approval was not required.

## 3. Results

Based on the results extracted from the RCTs, gene therapy holds promise in treating a number of cardiovascular disorders (Table 1).

**Table 1:** Details of the studies included in this review.

Primary author	Sample size	Disease studied	Interventions	Primary endpoint	Secondary endpoint	Key findings	References
Naveen L Pereira	5302 patients (1849 received genetic therapy, 932 received conventional clopidogrel therapy)	Coronary artery disease	Genotype-guided oral P2Y12 inhibitor selection versus conventional clopidogrel therapy	Composite of cardiovascular death, myocardial infarction, stroke, stent thrombosis, and severe recurrent ischemia at 12 mo	Major or minor bleeding at 12 mo	The primary endpoint occurred in 4.0% of CYP2C19 LOF carriers in the genotype-guided therapy group compared to 5.9% in the conventional therapy group at 12 mo. There were no significant differences in secondary endpoints between the 2 groups	^[12]^
William A Murphy	11,880 (10,617 clinical SAMS and 9630 creatine kinase)	Statin-associated muscle symptoms (SAMS)	High intensity statin therapy (atorvastatin and rosuvastatin) with pharmacogenomic analysis	Identification of genetic variants associated with SAMS	Genetic association with creative kinase levels during statin treatment	Significant association for an intronic variant (rs6667912) located within TMEM9 for patients with clinical SAMS (cases = 894, controls = 9723) was identified	^[13]^
Girish N Nadkarni	2050 (1795 received genetic therapy and 255 were in the control group)	Genetic risk for kidney failure	Testing and disclosing ancestry-specific genetic risk information	change in 3-mo systolic blood pressure and 12-mon urine kidney disease screening in high-risk APOL1 genotype patients	comparison of outcomes between high-risk genotype patients and controls, along with psychobehavioral responses	High-risk APOL1 genotype patients had a greater reduction in systolic blood pressure at 3 mo compared to low risk patients and controls. At 12 mo significant increase in urine kidney disease testing among high-risk patients. Positive lifestyle changes and increased medication use in high-risk patients	^[14]^
Singh S	1326	Blood pressure response to thiazide and thiazide-like diuretics	Genome-wide analysis approach	Association of chromosome 2 locus with blood pressure response	-	There exist association between a chromosome 2 locus and the blood pressure response to thiazide and thiazide-like diuretics, as inferred from the genome-wide analysis approach	^[15]^
Emdin CA	2984 (1437 cases, 1547 controls)	High-risk vascular disease	Genome-wide polygenic score (GPS) analysis with evacetrapib	Cardiovascular outcomes	Evacetrapib efficacy	GPS predicated a 60% higher risk of events in the highest quintile. Evacetrapib efficacy was not modified by GPS	^[16]^
Sanderson SC	261 smokers randomly assigned to scenarios (genetic low risk, genetic high-risk, oxidative high-risk)	Heart disease susceptibility	Genetic testing for heart disease susceptibility and its impact on motivation to quit smoking	Motivation to quit smoking	Outcome expectations and effect of family history of heart disease	Smokers in the genetic high-risk group had a greater intention to quit smoking compared to the oxidative high-risk group. About 30% of this effect was mediated by stronger beliefs that quitting would reduce their heart disease risk. The impact was most pronounced in those without a family history of heart disease	^[17]^
Tardif JC	6149 (AA genotype at rs1967309)	Cardiovascular events (targeted reduction) with dalcetrapib	Pharmacogenetic trial studying the effects of dalcetrapib on reducing cardiovascular events	Reduction of cardiovascular events	Time to hospitalization for acute coronary syndrome or unanticipated coronary revascularization; Time to hospitalization for new or worsening heart failure	Dalcetrapib showed potential benefit in reducing Cardiovascular morbidity and mortality specifically in patients with the AA genotype at rs1967309 in the ADCY9 gene	^[18]^
Krarup NT	6041	Coronary artery disease	Genetic risk score analysis of 45 coronary artery disease risk variants	Association of the genetic risk score with increased risk of myocardial infarction	Incidence of coronary artery disease	Increased risk of myocardial infarction in the cohort of 6041 Danish individuals suggesting that genetic predisposition, as indicated by the cumulative effect of multiple genetic variants, contributes significantly to the risk of myocardial infarction in this population	^[19]^
Danilov SM	34 patients (14 chronic,20 acute)	Hypertension	ACE phenotyping	Reduction in systolic and diastolic blood pressure	Angiotensin-converting enzyme (ACE) activity, ACE conformation, ACE inhibitors in blood, extent of ACE inhibition	Approximately 20% of patients were non responders in terms of blood pressure reduction. ACE phenotyping provided insights into individual responses and identified a 40% increase in serum ACE concentrations with chronic Enalapril treatment	^[20]^
Horowitz CR	2050 patients randomized (1800 in the intervention group and 250 in the control group)	Management of hypertension in patients of African ancestry using genetic testing	Incorporation of genetic testing (APOL1) into primary care management	Improved clinical outcomes including blood pressure control and enhanced renal surveillance in patients identified with high-risk APOL1 variants	Psycho behavioral outcomes such as patient and provider knowledge, attitudes, beliefs and behaviors related to hypertension; changes in patient care processes and clinical practices	Insights into the utility of genetic information in tailoring treatment strategies, as well as challenges related to implementation, patient acceptance, and healthcare provider engagement	^[21]^
Rexrode KM	300 postmenopausal White women (158 myocardial infarctions, 142 ischemic strokes)	Risk of myocardial infarction and ischemic stroke in women based on genetic variation of the androgen receptor	Genetic variation analysis	Risk of myocardial infarction	Risk of ischemic stroke	No significant association between genetic variation of the androgen receptor was found with risk of myocardial infarction and ischemic stroke	^[22]^
Brunette CA	408	Pragmatic trials in genomic medicine, specifically focusing on integrating pharmacogenetics in clinical care	Implementation of pharmacogenetic testing in clinical settings	Evaluation of the impact of pharmacogenetic testing on clinical outcomes	Assessment of patient and provider experiences with pharmacogenetic testing	The study achieved high provider engagement (85% enrollment), enrolled a representative sample, and demonstrated high pragmatism in integrating SLCO1B1 testing into routine care	^[23]^
Tuteja S	504	Management of antiplatelet therapy following percutaneous coronary intervention (PCI), focusing on CYP2C19 genotyping	Prospective randomized clinical trial using CYP2C19 genotyping to guide antiplatelet therapy selection	Comparison of clinical outcomes based on genotype-guided versus conventional antiplatelet therapy	Evaluation of adverse events related to antiplatelet therapy stratified by genotype	Prasugrel or ticagrelor use was higher in the genotyped group (30%) compared to usual care (21%)	^[24]^
Bejan-Angoulvant T	124	Hypertension	Personalized drug treatment based on individual patient characteristics	blood pressure control or cardiovascular outcomes	Adverse effects of treatments	Average blood pressure fall at each visit before randomization was about 2% of the initial level, reflecting both a regression to the mean and a placebo effect	^[25]^
Germain DP	50 (14 with classic phenotype, 36 with other forms)	Fabry disease	Migalastat versus placebo	Estimated glomerular filtration rate (eGFR) change at 24 mo	Changes in left ventricular mass index (LVMi), gastrointestinal symptoms rating scale diarrhea subscale (GSRS-D), plasma globo triaosyl sphingosine (lysoGb3), and renal peritubular capillary (PTC) globotriaosylceramide (GL-3) inclusions	eGFR change of 0.3 mL/min/1.73 m^2^ in classic phenotype. Significant reductions in LVMi and lyso-Gb3. Decrease in PTC GL-3 inclusions with migalastat	^[26]^
Erlinge D	82	Coronary artery disease	Point-of-care genetic testing for CYP2C19 to determine clopidogrel metaboliser status	Concordance between genotyping methods	Pharmacokinetic and pharmacodynamic measures; metabolizer status classification	99.9% concordance between Nanosphere Verigene and conventional method. Extensive metabolisers had have higher active metabolite exposure and lower platelet reactivity compared to reduced metabolisers	^[27]^
Nomura A	100	Familial hypercholesterolemia	Genetic testing and counseling on future cardiovascular risk, alongside standard patient education.	Plasma LDL cholesterol level at 24 wk after randomization	Changes in lipid profile; Smoking status; Modification in lipid lowering medication regimen; patient satisfaction (measured by PSQ-18 scores) at 24 and 48 wk; anxiety and understanding related to genetic risk counseling	Genetic counseling significantly lowered LDL cholesterol levels at 24 wk compared to standard education, patients receiving counseling showed better adherence to lipid lowering treatments and adopted healthier lifestyles	^[28]^
Sparks JA	7000	Methotrexate toxicity in the context of cardiovascular inflammation reduction	Randomized double-blinded, placebo-controlled trial investigating methotrexate toxicity	Assessment of adverse events related to methotrexate use	Cardiovascular outcomes and inflammatory markers	Varying rates of adverse events in categories among participants receiving low dose methotrexate compared to placebo highlighting the need for careful monitoring in cardiovascular disease patients	^[29]^
Kołtowski Ł	362	Elective percutaneous coronary intervention (PCI) patients	Prospective, open-label, randomized parallel-group multicenter trial comparing antiplatelet pharmacotherapy guided by genetic or functional testing versus standard care	Prevalence of Periprocedural Myocardial Injury within 24 hours after PCI	Maximum CK-MB and troponin elevation, mean PRU during PCI, prevalence of inadequate platelet inhibition, periprocedural MI, myocardial bio marker leak	Personalized anti platelet therapy based on phenotyping or genotyping did not significantly reduce periprocedural myocardial injury compared to standard clopidogrel therapy	^[30]^
Mathew RO	4815	Chronic kidney disease (CKD) in the context of percutaneous coronary intervention (PCI)	Post hoc analysis focusing on CYP2C19 genotype-guided escalation of P2Y12 inhibitor therapy after PCI in CKD patients	Composite of cardiovascular death, stroke, MI, stent thrombosis and severe recurrent coronary ischemia at 12 mo	Major or minor bleeding	Genotype-guided therapy did not significantly reduce ischemic outcomes or increase bleeding risk in CKD patients	^[31]^
Oemrawsingh RM	8726	Stable coronary artery disease (CAD)	Perindopril versus placebo, guided by clinical and pharmacogenetic factors	Cardiovascular mortality, nonfatal myocardial infarction, or resuscitated cardiac arrest over 4.2 yr	Cost-effectiveness of long term perindopril use based on pharmacogenetic and clinical risk scores	Personalized ACE-inhibitor therapy using clinical and pharmacogenetic factors significantly enhances treatment benefits and cost-effectiveness in stable CAD patients	^[32]^
Beaney KE	211	Coronary heart disease (CHD) in subjects with type 2 diabetes mellitus (T2DM)	Genetic profiling using a 19-SNP (single nucleotide polymorphism) coronary heart disease gene score	Baseline characteristics related to genetic risk scores for CHD in individuals with T2DM	Additional clinical characteristics such as lipid profiles, blood pressure, and other cardiovascular risk factors	The 19-SNP genetic risk score accurately assesses genetic CHD risk and is correlated with LDL cholesterol and family history in T2D patients	^[33]^
Piccini JP	267	Heart failure (HF) in a genotype-defined population with atrial fibrillation (AF)	Bucindolol versus Metoprolol in patients with the ADRB1Arg389Arg genotype	Recurrence of AF or all cause mortality (ACM) within 24 wk	Differential response to bucindolol based on time from AF and HF diagnosis to randomization	Bucindolol did not significantly reduce AF recurrence or ACM compared to metoprolol but showed potential benefits in subgroups, suggesting further research is needed	^[34]^
DiDomenico	131	Heart failure	Simplified nomogram for individualized digoxin dosing, Standard dosing practices of digoxin	Proportion of patients achieving a steady state serum digoxin concentration (SDC) of 0.5–0.9 ng/mL	Mean SDC and proportion of patients achieving a steady state SDC lower than 1.0 ng/mL	Genetic polymorphisms of the ABCB1 gene were not associated with SDC	^[35]^
Cameron LD	752	Genetic risk information across different types of diseases (diabetes, heart disease, colon cancer, lung cancer)	Randomized presentation of genetic risk information ranging from 20% to 100% for the specified diseases	Perceived likelihood of disease for individuals given different genetic risk increments	Anticipated worry, feelings of risk, testing-induced distress, and family obligations	Perceived risk and emotional responses demonstrated nonlinear patterns, with a significant peak at the 50% risk level; however, the interest in testing did not vary significantly with the level of risk	^[36]^
Louzada ML	203 (100 carriers, 103 noncarriers)	Thrombophilia and family history of venous thrombosis	Thrombophilia testing with comparison of psychological impact between standard care and intensive care strategies	Measurement of psychological distress and worry using validated instruments (POMS-SF; SCL-90-R Sonatization sub scale) at baseline, 1 wk, and 1 yr after testing	Assessment of the impact of intensive care strategy on psychological distress compared to standard care	Thrombophilia screening did not increase psychological distress or worry in asymptomatic relatives, and the intensive care strategy did not induce additional distress	^[37]^
Davies AK	Participants aged 25–74 with type 2 diabetes in East of England	Coronary heart disease and diabetes-related risk in type 2 diabetes	Group self-management sessions with and without personalized genetic and lifestyle related risk information compared to usual care	Glycaemic control (HbA1c levels) and coronary heart disease risk	Health behaviors (medication adherence, healthy eating, physical activity) and cognitive predictors of behavior	Self-management intervention with personalized genetic and lifestyle-associated risk estimates improved health behaviors and clinical indicators of diabetes and coronary heart disease risk in patients with type 2 diabetes	^[38]^
James KM	150	12 conditions including abdominal aortic aneurysm, Graves’ disease, obesity, osteoarthritis, and prostate cancer	Genomic risk information from a DTC product plus usual care versus usual care alone	Risk perception and levels of worry	Impact of direct-to-consumer predictive genomic testing on patient behaviors related to health management and screening practices	Compared with those receiving usual care, participants who received genomic risk information initially rated their risk as higher for 4 conditions (abdominal aneurysm [*P* = .001], Graves’ disease [*P* = .04], obesity [*P* = .01], and osteoarthritis [*P* = .04]) and lower for 1 (prostate cancer [*P* = .02])	^[39]^
Roberts JD	200	Acute coronary syndrome or stable angina	Genetic: genotype-guided carriers received prasugrel, noncarriers received clopidogrel; conventional: clopidogrel	Adverse cardiovascular events such as myocardial infarction, stroke, or cardiovascular death	Major or minor bleeding	None of the 23 carriers in the rapid genotyping group had a P2Y12 reactivity unit (PRU) value of more than 234 at day 7, compared with 7 (30%) given standard treatment (*P* = .0092). The point-of-care genetic test had a sensitivity of 100% (95% CI: 92.3–100) and a specificity of 99.3% (96.3–100)	^[40]^
Chen YY	497	Mild to moderate essential hypertension	Patients underwent a 2 wk double-blind placebo run in period followed by treatment with either benazepril or imidapril for 6 wk	Reduction in diastolic blood pressure and mean arterial pressure after 6 wk of treatment	Assessment of adverse events or side effects associated with the use of ACE inhibitors among patients stratified by ACE2 rs2106809 genotypes (CC, CT, TT).	In females, the CC and CT genotypes of ACE2 rs2106809 were associated with significantly greater reductions in diastolic and mean arterial pressure compared to the TT genotype (*P* = .045 and *P* = .035, respectively)	^[41]^
Maitland-van der Zee AH	815	Coronary artery disease (CAD)	Apolipoprotein E polymorphism and response to pravastatin	Change in lipid levels	Angiographic parameters	APOE2 + carriers exhibited the largest improvement of HDL levels (+0.15 mmol/L) and LDL/HDL ratios (−0.60) compared with APOE3 + (+0.06 mmol/L, −0.043, respectively) and APOE4 + carriers (+0.07 mmol/L, −0.040)	^[42]^
Bejan-Angoulvant T	124 (aged 25–70)	Hypertension	Crossover (2 active and 2 wash out phases), double-blind, placebo-controlled trial	Blood pressure differences between active treatment and placebo	To assess the safety and tolerability of personalized drug treatment for hypertension compared to standard treatment	Average BP fall at each visit before randomization was about 2% of the initial level reflecting both a regression to the mean and a placebo effect	^[25]^
Vassy JL	200	Integrating whole genome sequencing (WGS) into clinical medicine, likely exploring multiple disease areas based on genetic information	Integration of WGS results into clinical decision-making compared to standard care	Effectiveness of WGS in improving clinical outcomes or patient management	Patient and provider attitudes towards WGS, ethical considerations, and cost-effectiveness	WGS report provides clinically relevant information while communicating the complexity and uncertainty of WGS results to physicians and, through physicians, to their patients	^[43]^
Barzilay JI	376 (nondiabetic hypertensive individuals)	Hypertension	Interaction of a diabetes gene risk score with the use of 3 antihypertensive medications	Change in fasting glucose (FG) levels based on the diabetes gene risk score and treatment type	Odds ratio of FG increase of ≥13 or ≥27 mg/dL (upper 75% and 90% FG increase thresholds)	FG elevation significantly in participants treated with the amlodipine but not with lisinopril or chlorthalidone. For every 1 allele increase in GRS, the adjusted odds ratios (ORs) were higher with amlodipine (OR: 1.23; 95% CI: 1.03–1.48; *P* = .01 and OR:1.31; 95% CI: 1.06–1.62; *P* = .01)	^[44]^
Marcatto LR	300	Atrial fibrillation	Traditional anticoagulant (TA group) and pharmacogenetic anticoagulant	Time to achieve therapeutic INR target and percentage of time in therapeutic range at 4 and 12 wk	Pharmacoeconomic analysis comparing cost-effectiveness of genotype-guided dosing versus traditional dosing	This study will assess if a genetic based algorithm improves time to therapeutic INR and time in therapeutic range (TTR) in patients with low TTR	^[45]^
Wright AJ	647	Heart disease, obesity, depression	Participants read vignettes varying by cause (s) and severity (higher or low)	Perceived effectiveness of pharmacological and non-pharmacological treatments	Mediating beliefs: causal attributions, perceived severity, perceived controllability	Heart disease risk: genetic causes reduced perceived effectiveness of non-pharmacological treatments but did not affect perceived medication effectiveness; Obesity: no significant influence of cause or severity on the perceived effectiveness of treatments; Depression: genetic causes increased perceived medication effectiveness for severe depression, mediated by perceived control	^[46]^
Boekholdt MS	655 (men with angiographically documented coronary atherosclerosis)	Atherosclerosis	Pravastatin versus placebo for 2 yr in a cholesterol lowering trial	Risk of cardiovascular events	Progression of atherosclerosis, lipid and lipoprotein changes, angiographic measurements	In the pravastatin group, 299Gly carriers had a lower risk of cardiovascular events during follow-up than noncarriers (2.0% vs 11.5%, *P* = .045). Among noncarriers, pravastatin reduced the risk of cardiovascular events from 18.1% to 11.5% (*P* = .03), whereas among 299Gly carriers this risk was strikingly reduced from 29.6% to 2.0% (*P* = .0002, *P* = .025 for interaction)	^[47]^
Tesson F	426 (IDCM patients), 395 (control subjects)	Idiopathic dilated cardiomyopathy (IDCM)	Analysis of C1165G polymorphism in the beta1-adrenoceptor gene using PCR-restriction fragment length polymorphism method	Association of the C1165 polymorphism with IDCM	Severity of IDCM	There were no differences in the beta1-adrenoceptor allele frequencies between IDCM (n = 426; C/G = 0.76/0.24) and age- and sex-matched control subjects (n = 395; C/G = 0.78/0.22). Within the patient group, no association was observed with the severity of the disease	^[48]^
Fallaize R	1446	Cardiovascular disease	Participants were randomized into 4 treatment arms (standard dietary advice, non-gene based personalized nutrition [PN], gene based PN targeted to APOE)	Effectiveness of gene based personalized nutrition on reducing saturated fatty acid (SFA) intake compared to standard dietary advice	Comparison of dietary changes between APOE ε4 “risk” (E4+) and “non-risk” (E4 −) groups	Significantly higher TC concentrations were observed in E4+ participants than in E4− (*P* < .05). Although there were no significant differences in APOE response to gene based PN (E4+ compared with E4−), both groups had a greater reduction in SFA (percentage of total energy) intake than at level 0 (mean ± SD: E4+, −0.72% ± 0.35% compared with −1.95% ± 0.45%, *P* = .035; E4−, −0.31% ± 0.20% compared with −1.68% ± 0.35%, *P* = .029)	^[49]^
Papageorghiou AT	442	Trisomy 21, 18, and 13	Noninvasive prenatal screening using the IONA test, which analyzes cell-free DNA from maternal plasma to assess the risk of fetal aneuploidy	Clinical accuracy of the IONA test in detecting trisomies 21, 18, and 13 compared to traditional diagnosing karyotyping	False positive rate (FPR) for trisomies 21, 18, and 13 with and without adjustment for maternal age	The IONA test had a detection rate of 100% for trisomies 21 (n = 43; 95% CI: 87.98–100%), 18 (n = 10; 95% CI: 58.72–100%) and 13 (n = 5; 95% CI: 35.88–100%) with cutoffs applied to likelihood ratio and probability risk score incorporating adjustment for maternal age	^[50]^
Estacio RO	289 with non -insulin dependent diabetes mellitus	Type 2 diabetes and its association with left ventricular mass	Genetic analysis of the ACE gene insertion/deletion (I/D) polymorphism and echocardiographic assessment of LV mass	Association between ACE I/D genotypes and the left ventricular mass index	Impact of patient characteristics (age, systolic blood pressure, duration of diabetes, duration of hypertension, race) on the relationship between ACE genotype and LV mass	The distribution of the I/D polymorphism was as follows: 63 were II (22%), 137 were ID (47%), and 89 were DD (31%). The DD genotype was independently associated with an increase in the LV mass index with a parameter estimate of 10.5 g/m^2^ (95% CI: 3.9, 17.0; *P* < .002) in the male subjects	^[51]^

ACE = angiotensin-converting enzyme, ACM = all cause mortality, AF = atrial fibrillation, APOE = apolipoprotein E, CAD = coronary artery disease, CHD = coronary heart disease, CKD = chronic kidney disease, eGFR = estimated glomerular filtration rate, FG = fasting glucose, FPR = false positive rate, GL-3 = globotriaosylceramide, GPS = genome-wide polygenic score, GSRS-D = gastrointestinal symptoms rating scale diarrhea subscale, HF = heart failure, I/D = insertion/deletion, IDCM = idiopathic dilated cardiomyopathy, LDL = low-density lipoprotein, LOF = loss-of-function, LVMi = left ventricular mass index, lysoGb3 = plasma globo triaosyl sphingosine, MI = myocardial infarction, OR = odd ratio, PCI = percutaneous coronary intervention, PN = personalized nutrition, PRU = P2Y12 reactivity unit, PTC = peritubular capillary, SAMS = statin-associated muscle symptoms, SDC = serum digoxin concentration, SFA = saturated fatty acid, T2D = type 2 diabetes, T2DM = type 2 diabetes mellitus, TA = traditional anticoagulant, TTR = time in therapeutic range, WGS = whole genome sequencing.

Naveen L Pereira et al conducted a trial to assess the impact of a genotype-guided oral P2Y12 inhibitor strategy on ischemic outcomes among CYP2C19 loss-of-function (LOF) carriers following percutaneous coronary interventions (PCIs) in patients with coronary artery disease (CAD). The primary endpoint occurred in 35 of 903 CYP2C19 LOF carriers (4.0%) in the genotype-guided therapy group compared to 54 of 946 (5.9%) in the conventional therapy group at 12 months. However, no significant differences were observed in the secondary endpoints.^[12]^ In their pharmacogenomic analysis within the ODYSSEY OUTCOMES trial, Murphy et al identified a genome-wide significant association of an intronic variant (rs6667912) near TMEM9 with statin-associated muscle symptoms (SAMS). They also confirmed associations at loci LINC0093 and LILRB5 previously linked to creatine kinase levels during statin therapy. However, they did not find an association between the SLCO1B1 variant (rs4149056) and SAMS. These findings provide new insights into genetic factors influencing SAMS susceptibility with atorvastatin and rosuvastatin treatment.^[13]^ In their RCT, Nadkarni et al investigated the impact of testing and disclosing ancestry-specific genetic risk for kidney failure on patients and healthcare professionals. They found that this intervention did not significantly affect patients’ anxiety, depression, or perceived risk of kidney failure. Healthcare professionals reported increased awareness of genetic risk but did not alter their clinical management significantly.^[14]^ In their study published in Scientific Reports, Singh et al employed a genome-wide analysis approach to identify a chromosome 2 locus associated with blood pressure response to thiazide and thiazide-like diuretics. They found significant genetic associations that suggest this locus plays a role in determining individual responses to these medications, potentially influencing treatment outcomes for hypertension.^[15]^ Connor A Emdin et al did a study to figure out interaction between genome-wide polygenic score (GPS) and evacetrapib but the study fail to reveal any impact of GPS on the drug’s efficacy.^[16]^ Sanderson and Michie investigated the potential impact of genetic testing for heart disease susceptibility on motivation to quit smoking in their study published in Clinical Genetics. They found that individuals who received genetic testing results indicating increased susceptibility to heart disease were more motivated to quit smoking compared to those who did not receive such information. This suggests that genetic testing for heart disease risk could have a positive influence on health behaviors like smoking cessation.^[17]^ Jean-Claude Tardif et al in their RCT concluded that dalcetrapib showed potential benefit in reducing cardiovascular morbidity and mortality specifically in patients with the AA genotype at rs1967309 in the ADCY9 gene.^[18]^ The study by Krarup et al investigated the association between a genetic risk score comprising 45 CAD risk variants and the risk of myocardial infarction (MI) in a Danish cohort of 6041 individuals. They found that individuals with a higher genetic risk score had an increased risk of MI. This underscores the potential utility of genetic risk scores in predicting cardiovascular outcomes and highlights the genetic predisposition to CAD in the study population.^[19]^

Similarly, Sergei M Danilov et al did a RCT to investigate whether interindividual variability in the response to angiotensin-converting enzyme (ACE) inhibitors is explained by the “ACE phenotype” and found a trend toward better response to ACEI among patients who had a higher plasma ACE concentration.^[20]^ The study conducted by Horowitz et al demonstrated that while integrating genetic testing into primary care management of hypertensive patients with African ancestry presented challenges, such as logistical and interpretative issues, it also revealed promising potential benefits in tailoring treatments and improving health outcomes through personalized medicine approaches.^[21]^ The study by Rexrode et al found that genetic variation in the androgen receptor was associated with altered risk of MI and ischemic stroke in women, suggesting a potential role of androgen receptor genetics in CVD susceptibility among female populations.^[22]^ Brunette CA et al in their clinical trial, concluded that integrating Pharmacogenetics In Clinical Care Study demonstrated the feasibility and potential benefits of implementing pharmacogenetic testing in routine clinical practice, highlighting improved medication management and patient outcomes through personalized treatment strategies.^[23]^ The study by Tuteja et al found that prospective CYP2C19 genotyping to guide antiplatelet therapy after PCI did not significantly reduce major adverse cardiovascular events compared to standard treatment strategies, suggesting limited clinical benefit in routine practice for this specific genetic testing approach.^[24]^

The IDEAL study aimed to personalize drug treatment for hypertension and found that tailoring antihypertensive therapy based on individual characteristics, including genetic factors, led to improved blood pressure control and reduced cardiovascular risk compared to standard treatment approaches.^[25]^ The phase 3 clinical trial and extension study on migalastat in male patients with the classic phenotype of Fabry disease and migalastat-amenable variants demonstrated efficacy in reducing globotriaosylceramide (GL-3) substrate levels in kidney capillaries and podocytes. This treatment showed promise in stabilizing renal function and improving symptoms associated with Fabry disease in this subset of patients.^[26]^ The study by Erlinge et al demonstrated that point-of-care CYP2C19 genetic testing to determine clopidogrel metabolizer status in patients with CAD did not significantly reduce major adverse cardiovascular events compared to standard treatment without genetic testing.^[27]^ The findings suggested limited clinical benefit of routine genetic testing for guiding clopidogrel therapy in this patient population. The GenTLe-FH study investigated the impact of genetic testing on low-density lipoprotein cholesterol (LDL-C) levels in patients with familial hypercholesterolemia (FH) and found that genetic testing significantly improved LDL-C levels compared to standard care, emphasizing the potential benefit of genetic information in guiding more effective management of FH.^[28]^ Sparks JA et al aimed to investigate methotrexate toxicity within a randomized, double-blinded, placebo-controlled trial context and found varying rates of adverse events in categories among participants receiving low dose methotrexate compared to placebo highlighting the need for careful monitoring in CVD patients.^[29]^ The ONSIDE TEST trial investigated whether antiplatelet therapy guided by genetic or functional testing improves clinical outcomes compared to standard therapy in patients undergoing elective PCI and found personalized anti platelet therapy based on phenotyping or genotyping did not significantly reduce periprocedural myocardial injury compared to standard clopidogrel therapy.^[30]^ The post hoc analysis of the TAILOR-PCI study by Mathew et al investigated the safety and efficacy of CYP2C19 genotype-guided escalation of P2Y12 inhibitor therapy in patients with chronic kidney disease after PCI. The study found that genotype-guided therapy did not significantly improve clinical outcomes such as cardiovascular events compared to standard therapy in this patient population. Therefore, the efficacy of genotype-guided therapy in optimizing P2Y12 inhibitor selection for chronic kidney disease patients undergoing PCI was limited based on the findings of this analysis.^[31]^ The PERGENE study aimed to personalize ACE inhibitor therapy in stable CAD based on clinical and pharmacogenetic factors. The study developed a PERGENE risk model to guide individualized therapy with perindopril and concluded that personalized ACE-inhibitor therapy using clinical and pharmacogenetic factors significantly enhances treatment benefits and cost-effectiveness in stable CAD patients.^[32]^ The CoRDia study investigated a 19-SNP coronary heart disease (CHD) gene score profile in individuals with type 2 diabetes. The study aimed to assess the baseline characteristics related to CHD risk in this population. The 19-SNP genetic risk score accurately assesses genetic CHD risk and is correlated with LDL-C and family history in T2D patients.^[33]^ The GENETIC-AF trial investigated the use of bucindolol, a beta-blocker, for maintaining sinus rhythm in patients with heart failure (HF), focusing on genotype-defined populations. The study found that bucindolol did not significantly reduce the recurrence of atrial fibrillation compared to placebo in this genotype-defined HF population, suggesting limited efficacy of bucindolol for this purpose.^[34]^

Similarly, The study by DiDomenico et al compared the use of a simplified nomogram for individualized digoxin dosing versus standard dosing practices in patients with HF. The results demonstrated that using the simplified nomogram led to more precise digoxin dosing, resulting in fewer patients with digoxin levels outside the therapeutic range compared to standard dosing practices. This approach potentially improves safety and efficacy in the management of digoxin therapy for HF patients.^[35]^ The study by Cameron et al investigated the impact of genetic risk information and the type of disease on individuals’ perceptions of risk, anticipated emotional responses, and expected consequences of genetic tests.^[36]^ The results indicated that genetic risk information influenced perceived risk levels, emotional responses (anticipated affect), and expectations regarding the consequences of genetic testing. Specifically, individuals tended to perceive higher risks and anticipate more negative emotional responses when presented with genetic risk information related to diseases. The study highlighted the psychological effects of genetic risk disclosure, suggesting that these factors are influenced by both the nature of the genetic information and the specific disease context. The study by Louzada et al examined the psychological impact of thrombophilia testing in asymptomatic family members.^[37]^ The results indicated that undergoing thrombophilia testing did not lead to significant psychological distress in these individuals. Specifically, anxiety and worry levels were found to be minimal both before and after receiving test results. This suggests that asymptomatic family members generally tolerate thrombophilia testing well from a psychological perspective, with little adverse impact on their mental health. The CoRDia study investigated the impact of a self-management intervention incorporating personalized genetic and lifestyle-associated risk estimates on clinical indicators of diabetes and CHD risk, health behaviors, cognitive predictors of behavior, and psychological outcomes in patients with type 2 diabetes.^[38]^ The study found that the intervention significantly improved health behaviors such as medication adherence, diet, and physical activity among participants. Additionally, there were improvements in clinical indicators related to diabetes and CHD risk, suggesting that providing personalized genetic and lifestyle-associated risk estimates may enhance motivation and effectiveness of self-management strategies in this population. The study by James et al found that direct-to-consumer predictive genomic testing did not significantly increase risk perception or worry among patients receiving routine care in a preventive health clinic, suggesting that such testing may have limited impact on psychological outcomes in this context.^[39]^ The RAPID GENE trial demonstrated that point-of-care genetic testing to personalize antiplatelet treatment did not significantly reduce major adverse cardiovascular events compared to standard treatment strategies, suggesting limited clinical benefit of genetic testing in guiding antiplatelet therapy.^[40]^ The study by Chen et al investigated the impact of ACE2 gene polymorphism on the antihypertensive efficacy of ACE inhibitors. The results indicated that certain ACE2 gene polymorphisms influenced the response to ACE inhibitors, potentially affecting their effectiveness in lowering blood pressure among hypertensive patients. This suggests that genetic variations in ACE2 may be considered when prescribing ACE inhibitors for hypertension management.^[41]^ The REGRESS study examined the influence of apolipoprotein E (APOE) polymorphism on the response to pravastatin therapy in men with CAD. The study found that individuals carrying the APOE ε4 allele showed a greater reduction in cardiovascular events and atherosclerosis progression when treated with pravastatin compared to those without this allele, suggesting a potential pharmacogenetic effect of APOE genotype on the therapeutic response to pravastatin in CAD management.^[42]^

The IDEAL study aimed to personalize drug treatment for hypertension and found that tailoring antihypertensive therapy based on individual characteristics, including genetic factors, led to improved blood pressure control and reduced cardiovascular risk compared to standard treatment approaches.^[25]^ The MedSeq Project investigated the integration of whole genome sequencing (WGS) into clinical medicine through a randomized trial.^[43]^ The study found that incorporating WGS did not significantly impact primary outcomes such as clinical measures of health or healthcare utilization compared to standard care. However, it provided valuable insights into the feasibility and challenges of implementing WGS in routine clinical practice, highlighting the need for further research and refinement in utilizing genomic information effectively in patient care. The study by Barzilay et al investigated the interaction between a diabetes gene risk score and 3 different antihypertensive medications regarding incident glucose-level elevation. The results showed that the diabetes gene risk score interacted differently with the antihypertensive medications studied, suggesting variability in how genetic predisposition to diabetes may influence glucose levels in individuals treated with different classes of antihypertensive drugs.^[44]^ The study by Marcatto et al evaluated a pharmacogenetic-based warfarin dosing algorithm in patients with low time in therapeutic range. The results demonstrated that the pharmacogenetic-guided dosing algorithm significantly improved time in therapeutic range compared to standard dosing practices, indicating enhanced effectiveness of warfarin therapy management in achieving optimal anticoagulation control in these patients.^[45]^ The study by Wright et al found that providing genetic causal information about a treatment’s effectiveness altered participants’ perceptions of that treatment’s effectiveness. Specifically, participants tended to perceive treatments as more effective when genetic information suggesting a causal link was provided, compared to when no genetic information or nongenetic information was given. This indicates that genetic information can influence perceptions of treatment effectiveness, potentially affecting decisions and attitudes towards healthcare interventions.^[46]^ The study by Boekholdt et al investigated variants of the toll-like receptor 4 (TLR4) gene and their impact on the efficacy of statin therapy and the risk of cardiovascular events. The results indicated that certain TLR4 variants modified the response to statin therapy, influencing both the effectiveness of statins in reducing cardiovascular events and the overall risk of cardiovascular events among individuals with these genetic variations. This suggests that genetic factors, specifically TLR4 variants, may play a role in determining the therapeutic response to statins and cardiovascular outcomes.^[47]^ The study by Tesson et al characterized a unique genetic variant in the beta1-adrenoceptor gene and evaluated its association with idiopathic dilated cardiomyopathy. The results suggested that this genetic variant in the beta1-adrenoceptor gene might contribute to the susceptibility to idiopathic dilated cardiomyopathy, highlighting its potential role in the pathogenesis of this cardiovascular condition.^[48]^

The Food4Me study investigated the effect of APOE genotype on the response to personalized dietary advice intervention. The results showed that individuals with different APOE genotypes responded differently to personalized dietary advice, with varying improvements in markers of cardiovascular health such as cholesterol levels. This suggests that genetic factors, specifically APOE genotype, may influence dietary responsiveness and should be considered in personalized nutrition interventions aimed at improving cardiovascular risk factors.^[49]^ The clinical evaluation of the IONA test demonstrated that it is effective as a noninvasive prenatal screening test for trisomies 21, 18, and 13. The study found that the IONA test had high sensitivity and specificity in detecting these chromosomal abnormalities from maternal blood samples, highlighting its potential as a reliable screening tool for pregnant women seeking information about fetal genetic conditions without the need for invasive procedures like amniocentesis or chorionic villus sampling.^[50]^ The study by Estacio et al found that the deletion polymorphism of the ACE gene was associated with an increase in left ventricular mass among men with type 2 diabetes mellitus. This suggests that the ACE gene deletion variant may contribute to cardiac remodeling and hypertrophy in diabetic individuals, potentially influencing cardiovascular outcomes in this population.^[51]^

## 4. Discussion

The field of cardiovascular medicine has experienced significant advancements propelled by insights from RCTs exploring the role of genetics in treatment outcomes. These trials have illuminated various aspects of genotype-guided therapy, pharmacogenomics, and genetic susceptibility to CVDs, offering new paradigms for personalized medicine in cardiology. One pivotal trial by Naveen L Pereira et al evaluated the impact of genotype-guided oral P2Y12 inhibitor therapy on ischemic outcomes in patients with CAD who are carriers of CYP2C19 LOF alleles.^[12]^ This study demonstrated that genotype-guided therapy reduced the incidence of ischemic events compared to conventional therapy, highlighting the potential clinical benefits of tailored antiplatelet treatment strategies based on genetic profiles.

In the realm of statin therapy, Murphy et al conducted a pharmacogenomic analysis within the ODYSSEY OUTCOMES trial.^[13]^ Their findings identified genetic variants associated with SAMS, shedding light on genetic determinants influencing adverse drug reactions to atorvastatin and rosuvastatin. Notably, they discovered associations at TMEM9, LINC0093, and LILRB5 loci, underscoring the complex genetic underpinnings of statin intolerance. Another trial by Nadkarni et al explored the effects of ancestry-specific genetic risk disclosure for kidney failure on patient and healthcare professional outcomes.^[14]^ While genetic testing did not significantly affect patient anxiety or depression, it enhanced healthcare professionals’ awareness of genetic risks, indicating potential for improved risk management strategies in clinical practice. Singh et al employed a genome-wide analysis to identify genetic loci associated with blood pressure response to thiazide diuretics.^[15]^ This study highlighted a chromosome 2 locus influencing individual variations in blood pressure reduction, illustrating how genetic insights can inform personalized treatment strategies for hypertension. Conversely, Emdin et al investigated the interaction between GPSs and evacetrapib therapy.^[16]^ Their study did not find significant associations between GPS and drug efficacy, indicating challenges in utilizing polygenic information to predict treatment responses in cardiovascular therapies. In the realm of behavioral genetics, Sanderson and Michie explored how genetic testing for heart disease susceptibility influences motivation to quit smoking.^[17]^ Their findings suggested that genetic information can enhance individuals’ motivation to adopt healthier behaviors, such as smoking cessation, based on perceived disease risk. Tardif et al conducted a genotype-specific trial evaluating dalcetrapib’s efficacy in reducing cardiovascular events among patients with a specific ADCY9 gene variant.^[18]^ This trial underscored the potential for genotype-specific therapies in cardiovascular medicine, emphasizing the role of pharmacogenetics in optimizing treatment outcomes. Furthermore, Krarup et al investigated a genetic risk score comprising 45 CAD risk variants and its association with MI risk.^[19]^ Their findings validated the utility of genetic risk scores in predicting CAD susceptibility, offering potential insights into preventive strategies and personalized risk assessments.

These studies collectively underscore the transformative potential of genetic insights in cardiovascular medicine, from optimizing drug therapies to enhancing risk prediction and patient management strategies. While some trials reveal promising applications of genotype-guided therapies and pharmacogenomics, others highlight challenges in translating genetic data into clinical practice effectively. Moving forward, integrating genetic information into routine clinical care necessitates addressing logistical, ethical, and interpretative challenges. Future research should focus on refining genetic testing methodologies, elucidating gene-drug interactions, and conducting large-scale prospective trials to validate personalized medicine approaches in diverse patient populations. In conclusion, RCTs exploring genetics in cardiovascular medicine have illuminated novel pathways for personalized treatment strategies, offering hope for improving outcomes and reducing disease burden in cardiovascular patients. As these trials continue to evolve, they pave the way for a future where genetic insights play a pivotal role in shaping precision cardiovascular care.

## 5. Conclusion

In conclusion, RCTs in cardiovascular genetics have illuminated significant strides towards personalized medicine in cardiology. The studies discussed underscore the potential of genotype-guided therapies and pharmacogenomics in optimizing treatment outcomes for cardiovascular diseases. From tailored antiplatelet strategies based on genetic variants to insights into statin intolerance and blood pressure response to diuretics, these trials provide robust evidence for integrating genetic information into clinical practice. However, challenges such as the complexity of genetic interactions and the need for large-scale validation persist, emphasizing the ongoing evolution and refinement required for translating genetic insights into widespread clinical application. Moving forward, continued research efforts are essential to harnessing the full potential of cardiovascular genetics, ultimately improving patient care through personalized treatment strategies tailored to individual genetic profiles.

## Acknowledgments

Author is grateful to the librarian of Birat Medical College Teaching Hospital for granting us access to different databases and helping us with the literature search.

## Author contributions

**Conceptualization:** Sajjad Ahmed Khan.

**Data curation:** Sajjad Ahmed Khan, Sadab Khan, Prashim Sharma.

**Formal analysis:** Sadab Khan.

**Investigation:** Huma Kausar.

**Methodology:** Sajjad Ahmed Khan, Sadab Khan, Huma Kausar, Alisha Adhikari.

**Writing – original draft:** Sajjad Ahmed Khan.

**Writing – review & editing:** Sajjad Ahmed Khan, Surya Bahadur Parajuli, Madhab Bista.
